# Impact of a right ventricular impedance sensor on the cardiovascular responses to exercise in pacemaker dependent patients

**Published:** 2005-07-01

**Authors:** Linnea Cook, Corey Tomczak, Edward Busse, John Tsang, Wladyslaw Wojcik, Robert Haennel

**Affiliations:** * Faculty of Kinesiology and Health Studies, University of Regina, Regina, Canada; † Regina General Hospital, Regina Qu’Appelle Health Region, Regina, Canada

**Keywords:** cardiac pacing, exercise responses, chronotropic reserve index

## Abstract

**Background:**

The evaluation of the heart rate (HR) response to exercise is important for the assessment of the rate response algorithm of sensor-controlled pacemakers. This study examined the effects of a right ventricular impedance sensor driven pacemaker on the cardiovascular responses to incremental exercise in pacemaker dependent patients.

**Methods:**

Twelve patients (70.5 ± 9.5 years; 5 Females: 7 Males) implanted with an Inos ^2+^ closed loop stimulation (CLS) pacemaker were compared to 12 healthy age and sex matched controls (70.6 ± 4.8 years). All subjects performed the chronotropic assessment exercise protocol (CAEP). Variables of interest included HR, cardiac output (Q), oxygen uptake (Vo_2_) and blood pressure (BP). Data were analyzed at rest, throughout exercise and during recovery. Furthermore, patient chronotropic responses were compared to a reference chronotropic response slope for aerobic exercise.

**Results:**

There were no differences between groups for HR or Q response throughout exercise. At peak exercise, Vo_2_ (mL.kg^-1^.min^-1^) was higher for the controls (p < 0.05). The patient chronotropic response slope was comparable to the CAEP reference slope from rest to both the anaerobic threshold (AT) and peak exercise. During recovery, no differences were observed between the groups for any parameters or for the HR decay slopes.

**Conclusion:**

Up to the anaerobic threshold, the right ventricular impedance sensor driven pacemaker delivered a pacing rate that contributed to an overall cardiovascular response similar to that observed in healthy age matched subjects.

## Introduction

Rate responsive pacemakers sense some physiological or non-physiological signal(s), and translate changes in that signal into a pacing rate that is appropriate for the metabolic demands of the patient. Most sensors, although coupled with a normal HR response through a surrogate parameter, do not directly measure cardiac dynamics. Since there is no direct “connection” between these surrogate parameters and the cardiovascular system, measurements are not always proportional, and are therefore, not always converted to an appropriate pacing rate for a given metabolic demand [1,2].

The Inos^2+^ CLS pacemaker (Biotronik, Inc.) employs closed loop stimulation (CLS) based on right ventricular impedance monitoring. This device monitors cardiac contraction dynamics by measuring localized intracardiac impedance signals. Changes in intracardiac impedance are evaluated, allowing for the assessment of changes in myocardial contractility during both diastolic and systolic phases of the cardiac cycle [3]. It’s reported that the CLS system integrates the pacemaker into the cardiovascular system, creating a negative feedback loop [4], such that the change in the pacing rate then has a direct effect on the sensed parameter (i.e., myocardial contractility). The purported advantage of closed loop pacing is that the change in HR influences the sensed surrogate parameter directly [5]. For this reason, the Inos^2+^ CLS system is theorized to perform well in terms of proportionality and response times when compared to a healthy sinus response.

The evaluation of the heart rate (HR) response to exercise is important for the assessment of the rate response algorithm of sensor-controlled pacemakers. The rate responsiveness should closely simulate the chronotropic responsiveness of a healthy heart. New sensor based pacing systems are commonly evaluated using a mathematical model of the normal chronotropic response to exercise described by Wilkoff et al [6]. Thus, the purpose of the present study was to examine the effect the Inos^2+^ CLS pacing system on the cardiovascular responses during exercise and to compare these responses with those from a healthy age matched control group.

## Materials and Methods

### Study Group

The subjects included 12 pacemaker patients and 12 healthy controls (Table I). Eight of the 12 pacemaker patients were completely chronotropically incompetent as defined by sinus bradycardia and block. Patients were recruited from a Pacemaker Clinic at a major city hospital and had previously been implanted with an Inos^2+^ CLS pacemaker (Biotronik, Inc.) a minimum of four weeks prior to commencement of the study. The control subjects were age and sex matched with no history or clinical presentation of cardiovascular and/or pulmonary disease. All subjects were ambulatory and able to perform a treadmill test and continued with their usual drug therapy during this study. The study complied with the Declaration of Helsinki and was approved by the Health Regions’ Research Ethics Board. All subjects provided written informed consent prior to entry into the study.

### Pacemaker Programming

The Inos^2+^ CLS pacemaker used a right ventricular impedance sensor to continually monitor the contractile state of the myocardium and converts this intrinsic information into an appropriate HR using the programmed lower and upper rates as endpoints. Specifically, the internal impedance sensor of the pacemaker measured the myocardial contractility eight times throughout each cardiac cycle to determine the contractility waveform. This new waveform was compared to a baseline waveform and the area defining the difference between the two waveforms was used to calculate the appropriate HR increase.

The pacemaker’s lower pacing rate (BR = basic rate) ranged from 45 to 60 beats.min^-1^, as determined from the patients’ clinical presentation. The pacemaker’s maximum sensor driven rate (MCLR = maximum closed loop rate) was set at 80 to 100% of the age-predicted maximum HR (calculated as 220 - age), depending on the patient’s daily activity, fitness level and disease status. If the patient had a history of exercise induced angina, the MCLR was set at least 10 beats.min^-1^ below the ischemic threshold.

### Exercise Testing

Following five minutes of seated rest, measurements were recorded for all parameters. Another two minutes of standing rest was incorporated to allow the pacemaker algorithm to set the baseline in a standing pre-exercise position. Following, each subject performed a graded exercise test on a treadmill (Trackmaster, model #215, JAS MFG, Carrollton, TX, USA). The chronotropic assessment exercise protocol (CAEP) was performed to peak capacity or until symptoms limited any further progression of exercise intensity [7]. The CAEP is a maximal treadmill protocol designed specifically for the evaluation of rate-responsive pacemakers [6,8]. This protocol begins at 1.5 METs (1 MET = 3.5 mL O_2_.kg^-1^.min^-1^) and consists of two-minute stages with small increments of approximately 1 MET per stage for the first 10 minutes, after which it increases by 2 to 3 METs per stage. This allows most patients to complete several stages of exercise and tests the chronotropic response to submaximal exertion that falls within the range of many activities of daily living [6].

### Heart Rate

A standard 12-lead ECG was recorded continuously using Merlin AM™ hardware (CardioComm Solutions, Inc., Victoria, BC, Canada). Following the test, beat-to-beat HR values were measured at 100 mm.sec^-1^ using GEMS^TM^ and a custom Annoexport program (CardioComm Solutions, Inc., Victoria, BC, Canada, 2000). In the patient group, the HR origin was identified (e.g., paced, sensed or NSR = normal sinus rhythm) and only paced beats were used for analyses. For each stage of exercise, the HR was determined using an average of five beats from the last 10 seconds of each minute. Peak HR was determined using the HR value measured in the last 10 seconds prior to test termination. Post-exercise rates were determined using the same measurement method at three minutes into recovery. Additionally, HR was measured beat-to-beat for the initial two minutes of recovery and 5-second averages were plotted against recovery time in order to generate HR decay slopes for the two groups.

### Metabolic Analysis

Oxygen uptake (Vo_2_) and carbon dioxide production (V_co2_) were recorded at rest, throughout exercise and during the three-minute recovery period. Expired gases were collected using the TrueMax 2400 metabolic measurement system (Parvo Medics Inc., Salt Lake City, UT, USA) and a Hans Rudolph 3813 heated pneumotach flow meter. The gas analysis system collected samples of oxygen (O_2_) and carbon dioxide (CO_2_) on a breath-by-breath basis and displayed 15-second averages to account for the variation typically present in breath-by-breath measurements. Because it has been suggested that the anaerobic threshold (AT) may be used as an objective measure of chronotropic function in pacemaker patients [9], AT was calculated from the metabolic data using the V-slope method, where AT is defined as the point in which the relationship between V_co2_ and Vo_2_ became non-linear [10,11]. Peak Vo_2_ was determined as the highest value calculated over a 30-second period [11]. Oxygen pulse (O_2_ Pulse) (mL O_2_.beat^-1^) was calculated as Vo_2_/HR for rest, each minute of exercise and minute three of recovery. This parameter is equal to the product of stroke volume (SV) and arterial-venous oxygen difference and was used as an indirect measure of SV [11].

### Hemodynamic Measurements

Stroke volume was determined using an electrical impedance device (Minnesota Impedance Cardiograph, Model 304 B; Surcom Inc., Minneapolis, MN, USA), a Hewlett Packard phonocardiogram (model 21050A) and a 3-lead ECG recorder. The Bernstein equation [12] was used to calculate cardiac output (Q) at rest, for each exercise stage and at three minutes post-exercise. Impedance cardiography has been widely used and validated as a non-invasive measure at rest and during exercise in post-myocardial infarct and pacemaker dependent patients with < 5% random error [13-15]. As well, Bernstein’s equation has become commonplace in impedance cardiography techniques as it has been shown to result in values consistent with previously developed equations [16]. Blood pressure (BP) was assessed using a mercury sphygmomanometer. Systolic (SBP) and diastolic blood pressure (DBP) was recorded at rest, for each exercise stage and during recovery. Mean arterial pressure (MAP) was calculated as DBP + 1/3 (SBP - DBP).

### Calculations

Work rate (WR) was calculated in watts (W) for each subject at each stage of exercise using the formula [17]:

WR (W) = weight (kg) x 9.8 (m.sec^-2^) x speed (m.sec^-1^) x sin ß

where weight was the subject’s weight (kg), 9.8 (m.sec^-2^) was the force of gravity, speed was the treadmill speed (m.sec^-1^) and sin ß was the vertical displacement for a given horizontal displacement

Heart rate to WR and Vo_2_ to WR ratios were calculated for each minute of exercise. Oxygen uptake and HR were displayed relative to WR and regression equations were calculated using lines of best fit. From this, the aerobic power slope was calculated as Vo_2_.kg^-1^.WR^-1^ and expressed in mL.kg^-1^.watt^-1^. The percent of HR reserve (%HRR) was calculated for each WR as:


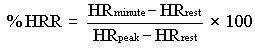


where HR_minute_ was the HR at each minute of exercise, HR_rest_ was the programmed BR or resting HR for patients and controls respectively and HR_max_ was the programmed MCLR or peak HR for patients and controls respectively.

Percent metabolic reserve (%MR) was calculated using MET levels measured with the gas analysis system as follows:


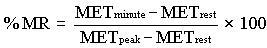


where MET_minute_ was the calculated MET level at each minute of exercise, MET_rest_ was the MET level at rest and MET_peak_ was the MET level at peak exercise for patients and controls.

The Chronotropic reserve index (CRI), defined as relationship between %HRR and %MR, was compared to the chronotropic response slope calculated by Wilkoff et al [6] for the CAEP. This response was evaluated from rest to peak exercise, rest to the AT and from the AT to peak exercise.

## Statistical Analysis

Data analysis was performed using the Statistical Package for Social Sciences (SPSS 9.0, Chicago, IL) program. Data are expressed as mean ± standard deviation (SD). A two-way repeated measures analysis of variance (ANOVA) was employed to investigate differences within and between groups. Data at rest, peak exercise and three minutes post-exercise were compared between groups using one-way ANOVA. Correlation-regression analysis was used to determine the relationship between Vo_2_, Q and HR for each group, as well as HR and VO_2_ to WR and %HRR to %MR. When regression lines were compared, a t-test was employed to test for a difference in slopes [18]. For all analyses, significance was set at p < 0.05 and 95% confidence intervals were calculated around several variables for purposes of comparison.

## Results

This study was completed on 12 patients (70 ± 9.5 years) and 12 age and sex matched controls (70.6 ± 4.8 years). There were no differences between the groups with respect to age, weight (patients = 77 ± 12 kg versus 71 ± 9 kg) or height (patients = 163 ± 12 cm versus 165 ± 12 cm). There were no significant differences in any of the parameters when seated versus upright rest were compared. Resting Vo_2_ (mL.kg^-1^.min^-1^) and DBP were higher (p < 0.05) in the control group (Table II).

### Exercise Performance

In the patient group, 11 of 12 tests were terminated due to volitional fatigue (one test was terminated due to angina). Six of the 12 patients exceeded their MCLR during the exercise test, with the pacemaker providing appropriate ventricular tracking. Non-paced HR data above the MCLR were excluded from analyses. For the control group, all tests were terminated due to volitional fatigue. The exercise duration was significantly (p < 0.05) shorter in the patient group (10.3 ± 2.8 minutes) versus the control group (12.5 ± 2.1 minutes).

### Exercise Responses

**Figure 1:**
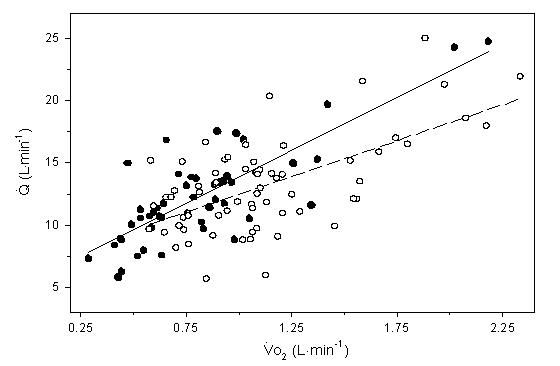
Regression lines showing the relationship between cardiac output (Q) and oxygen uptake (Vo_2_) during submaximal exercise for paced patients (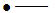) Q= 8.6    (Vo_2_) + 5.3, r = 0.81, p < 0.001; and healthy controls (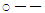) Q= 5.8 (Vo_2_) + 6.7, r = 0.62, p < 0.001. There were no significant differences in the slopes between groups (p > 0.05)

**Figure 2:**
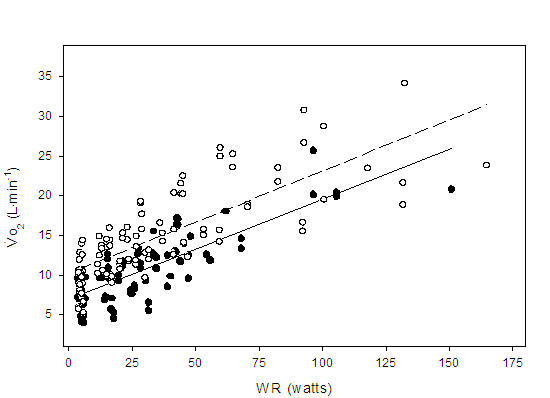
Regression lines showing the relationship between oxygen uptake (Vo_2_) and work rate (WR) during submaximal exercise for paced patients (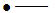) Vo_2_ = 0.13 (WR) + 7.0, r = 0.81, p < 0.001; and healthy controls (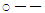) Vo_2_ = 0.13 (WR) + 10.2, r = 0.78, p < 0.001. There were no significant differences in the slopes between groups (p > 0.05)

**Figure 3:**
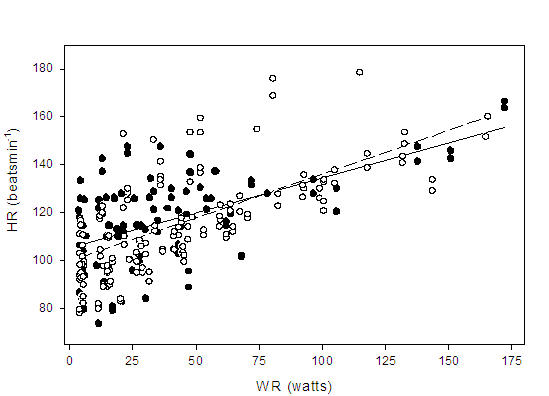
Regression lines showing the relationship between heart rate (HR) and work rate (WR) from rest to peak exercise for paced patients (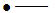) HR = 0.29 (WR) + 105.4, r = 0.55, p < 0.001; and healthy controls (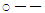) HR = 0.36 (WR) + 99.8, r = 0.67, p < 0.001. There were no significant differences in the slopes between groups (p > 0.05)

The mean correlation coefficient for HR:Vo_2_ was calculated to be r = 0.81 and 0.90 for the patient and control group respectively (p < 0.001). There was however, a wide range of slopes within each group, which may be attributed to differences in age, peak functional capacity or resting HR between individuals [6,19]. Additionally, there was a significant correlation (r = 0.71, p < 0.001) for Q:Vo_2_ for the entire subject pool (Figure 1). When Vo_2_ (mL.kg^-1^.min^-1^) was plotted against WR, a positive linear correlation was observed for patients (r = 0.81) and controls (r = 0.78) (p < 0.05). Throughout exercise, the line of best fit defining the relationship between Vo_2_ and WR was consistently higher in the controls (Figure 2) (p < 0.001). However, a t-test indicated no difference between the slopes of the two lines (p > 0.05). The aerobic power slopes were calculated to be 0.13 ± 0.03 and 0.13 ± 0.04 mL.kg^-1^.watt^-1^ for patients and controls respectively. The HR:WR ratio was also calculated for each subject at each minute of exercise. When these ratios were compared, no differences were found between the patients and controls (Figure 3). The slopes for HR to WR ratios were 0.29 ± 0.14 and 0.36 ± 0.11 for patient and control groups respectively.

The patient group reached their AT in 8.6 ± 2.9 minutes and the controls reached their AT in 9.1 ± 4.3 minutes. A t-test indicated no between group differences in time to reach AT or AT expressed as a percent of peak Vo_2_. Further, Vo_2_ at AT was comparable for patients (0.88 ± 0.34 L.min^-1^) and controls (1.17 ± 0.43 L.min^-1^). The peak Vo_2_ (mL.kg^-1^.min^-1^) was significantly higher in the control subjects (p < 0.05). At peak exercise, no differences were observed between the groups for any other parameters (Table II).

### Chronotropic Reserve Index

The chronotropic reserve index (CRI) was defined as the relationship between %HRR and %MR. For the healthy control group, the slope from rest to peak exercise was 0.83 ± 2.02 and the y-intercept was 4.50 ± 13.0% with a correlation coefficient of r = 0.82 (p < 0.001). These finding were comparable to the Wilkoff CRI slope al [6] which was reported to be 0.94 ± 0.12 with a y-intercept of 4.58 ± 7.7%. When data from rest to AT were analyzed, the slope was found to be 0.83 ± 0.26 and the y-intercept was 2.4 ± 11.6% with a correlation coefficient of r = 0.82 (p < 0.001). The CRI correlation from AT to peak exercise was not significant (p > 0.05).

**Figure 4:**
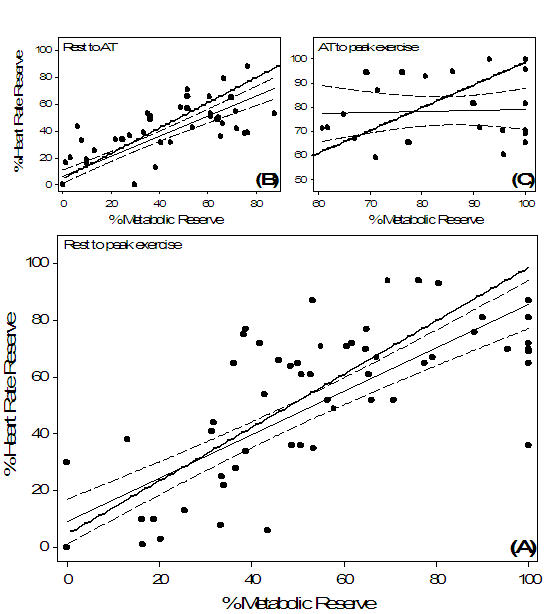
Chronotropic response index (CRI) for paced patients (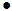, regression 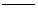 and 95% confidence interval 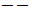) versus Wilkoff’s[6]. model (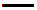) for healthy subjects. The CRI was calculated from (**A**) rest to peak exercise: %HRR = 0.77 (%MR) + 8.98, r = 0.81, p < 0.001 and (**B**) rest to anaerobic threshold (AT): %HRR = 0.75 (%MR) + 6.13, r = 0.85, p < 0.001. There was no difference in the slopes between patients and the healthy reference response model from rest to peak exercise (**A**) and from rest to AT (**B**) (p > 0.05). The regression from AT to peak exercise (**C**) was not significant (p > 0.05) and was therefore not compared with the reference line

For the patient group, the CRI slope from rest to peak exercise was 0.77 ± 0.24 and the y-intercept was 8.95 ± 13.8% with a correlation coefficient of r = 0.81 (p < 0.001). When data from rest to AT were analyzed, the slope was found to be 0.75 ± 0.21 and the y-intercept was 6.13 ± 8.7% with a correlation coefficient of r = 0.85 (p < 0.001) (Figure 4). The CRI from rest to peak exercise and from rest to AT was then compared to the findings of Wilkoff et al [6]. The slopes from both analyses fell within the 95% confidence intervals (0.94 ± [2 x 0.12] = 0.7 to 1.18) of healthy subjects who performed the CAEP in the original study [6]. Similarly, the y-intercepts were within 2 SD of the mean for normal subjects (^-1^0.82 to 19.98). The CRI correlation data from AT to peak exercise was not significant (p > 0.05) for paced patients and as a result, was not compared to the Wilkoff CRI slope [6] (Figure 4).

### Exercise Recovery

**Figure 5:**
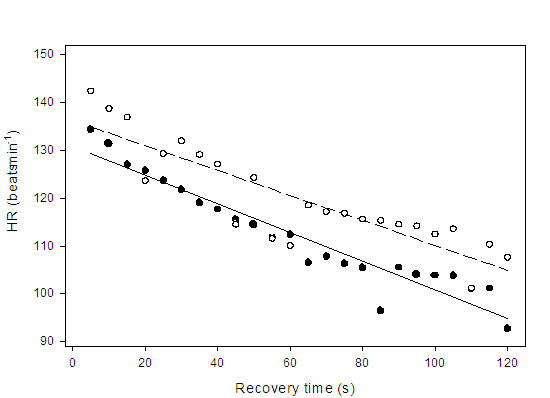
Regression lines showing the relationship between heart rate (HR) decay and recovery time from peak exercise (Time 0) through the first 120 seconds of recovery for paced patients (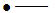) HR = -0.30 (recovery time) + 130.9, r = 0.96, p < 0.001; and healthy controls (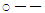) HR = -0.26 (recovery time) + 136.2, r = 0.87, p < 0.001. Data points represent 5 sec averages. There were no significant differences in the slopes between groups (p > 0.05)

No group differences were observed for any parameters during recovery. By minute three, all parameters had decreased (p < 0.05) relative to peak values, with the exception DBP (Table II). When HR decay was calculated, there was a significant (p < 0.001) correlation between the decrease in HR and recovery time for patients (r = 0.96) and controls (r = 0.88) (Figure 5). The slope of HR decay for the patients was -0.30 ± 0.07 and the y-intercept was 130.9 ± 4.8 beats.min^-1^. The slope of HR decay for the healthy controls was -0.26 ± 0.11 and the y-intercept was 136.3 ± 7.6 beats.min^-1^. When the HR decay slopes were compared, no differences (p > 0.05) were observed between groups.

## Discussion

The purpose of this study was to assess the impact of a right ventricular impedance sensor driven pacemaker on cardiovascular responses to exercise and to compare these responses with those from a healthy age matched control group. At rest, throughout exercise and at peak exercise, Vo_2_ (mL.kg^-1^.min^-1^) was consistently higher in the control group (Figure 2). However, no between group difference was observed in the slopes of the lines defining the overall relationship between Vo_2_:WR, suggesting the responses were similar for patients and controls. The patients in our study exercised for 10 ± 2.8 minutes while attaining a peak Vo_2_ of 15 ± 9 mL.kg^-1^.min^-1^. These results were similar to those reported in two previous studies on pacemaker patients [20,21] but were lower than those observed in younger subjects [6] and the healthy elderly [22]. The group of young, healthy subjects studied by Wilkoff et al.[6] reached a peak MET level of 11.3 ± 2.4 during the CAEP. In a study by Page et al [22], healthy elderly subjects exercised for 14.7 ± 2.9 minutes and reached peak Vo_2_ of 28.7 mL.kg^-1^.min^-1^ (8.2 METs).

In a study by Freedman et al [23], the performance of paced patients closely resembled that of healthy subjects reaching stage 8 of the CAEP with a predicted peak MET level of 12.1. Dailey et al [24] also reported a higher peak Vo_2_ (1.61 ± 0.45 L.min^-1^) for paced patients (MV, dP/dt_max_ or SaO_2_ sensors) than was observed in the present study (1.1 ± 0.7 L.min^-1^). Similarly, Meine et al [9] reported a peak Vo_2_ of 24 ± 4 mL.kg^-1^.min^-1^ in a sample of pacemaker dependent patients. Possible reasons for the lower peak MET level achieved by the patients in our study could include the group’s advanced age [25] or the MCLR having been programmed at less than the age-predicted maximum HR [21].

The peak Vo_2_ observed for our patient group was consistent with those reported previously [26,27]. Studies by Leung et al [27] and Haennel et al [26] had paced patients perform maximal exercise while programmed in one of three sensor modes (activity, QT interval and dual). In these studies, the peak Vo_2_ reported for activity and dual sensor settings were consistent what we observed in our patient group. Further, Carmouche et al [21] reported treadmill exercise duration of 10.6 minutes in pacemaker patients, but Vo_2_ was reported in mL.min^-1^ without information on patients’ weight, thus precluding further comparison with our data. Kay [20] found the maximum achieved Vo_2_ of pacemaker patients during the CAEP to be 13.2 ± 4.1 mL.kg^-1^.min^-1^ (3.7 ± 1.2 METs).

The paced patients reached AT in 8.6 ± 2.9 minutes (~ 73% of peak Vo_2_) and the healthy controls reached AT in 9.1 ± 4.3 minutes (~ 69% of peak Vo_2_). These results compare favorably with the AT achieved at 75% of maximal Vo_2_ as reported by Carmouche et al [21], but were higher than reported in another study of normal subjects. Page et al [2] found that healthy elderly subjects reached AT at 65% of maximal Vo_2_ during CAEP exercise testing. Wasserman et al [11] states that the mean AT can be expected to occur between 55 and 65% of maximal Vo_2_ for individuals aged 40 to 70 years. The ratio of AT to maximal Vo_2_ increases as a function of age, with this value being higher for women than men. For the mean age of our study group (70 years), we calculated the 95% confidence intervals of AT / maximal Vo_2_ as being 47 to 69% for men and 54 to 76% for women. Therefore, the AT / maximal Vo_2_ ratio of 72% from our study was within 2 SD of the mean of healthy 70-year-old subjects.

There were no differences in the AT or the Vo_2_ at AT between our two groups. Given that the AT represents a physiological breakpoint in the various cardiopulmonary parameters during exercise, we calculated the slopes below and above AT. The calculated aerobic power slopes for the patients and controls, from rest to AT, were 0.13 ± 0.03 mL.kg^-1^.watt^-1^ and 0.13 ± 0.04 mL.kg^-1^.watt^-1^ respectively. These slopes were similar to what was reported by Lewalter et al [28] for healthy men and women (0.22 ± 0.09 mL.kg^-1^.watt^-1^), suggesting an appropriate aerobic response for our patients up to AT. In the study by Lewalter et al [28], a 32% reduction in the slope above AT was reported (0.15 ± 0.07 mL.kg^-1^.watt^-1^), whereas in the present study, the slope above AT was found to be 0.05 ± 0.20 mL.kg^-1^.watt^-1^ equivalent to a mean decrease of approximately 38%. These observations are consistent with previous results demonstrating that increases in Vo_2_ above the AT become smaller than the increases in WR [29].

The observed HR:WR slopes for our patients (0.29 ± 0.14 beats.min^-1^.watt^-1^) and controls (0.36 ± 0.11 beats.min^-1^.watt^-1^) were comparable to a slope reported for healthy men (0.33 ± 0.14 beats.min^-1^.watt^-1^) [28]. The present data suggests that the pacemaker yielded a HR response consistent with that seen in a healthy population for increments in metabolic demand. Further, our findings are consistent with a study by Malinowski [2] who reported that a CLS group demonstrated a HR response that was consistently similar to a control group across a variety of tasks including slow and fast walking, climbing and descending stairs, arm movements and cognitive challenges.

From rest to both AT and peak exercise, the patient CRI slope was found to be comparable to the CAEP reference slope [6] (Figure 4, A and B), suggesting that the CLS pacemaker response was proportional to increments in exercise load. At workloads above the AT, the CRI slope for the patients indicated that %HRR did not statistically correlate with %MR, although a decrease in slope was apparent (see Figure 4, C). This reduction in the slope above AT may have contributed to the lower rest to peak exercise correlation coefficient (r = 0.81) when compared with rest to AT data (r = 0.85). Poor correlations between HR and WR above the AT have been previously reported with other sensors [27,28]. Given that a low CRI correlation above AT was also observed in our control group suggests that either a reduction in CRI slope above AT is a normal physiologic response or, the methodological analysis is not sensitive enough to elucidate the underlying physiologic phenomenon. Using healthy young subjects, researchers [30] have demonstrated that the HR slope in relation to WR does deflect downward at approximately 90% of maximal HR or 70% of maximal Vo_2_. It has been further demonstrated that this phenomenon corresponds with the AT [31,32]. Thus, if the current method of analysis for chronotropic responses above AT was appropriate, then the present findings would further implicate the AT as an important signaling mechanism to consider during rate response pacing.

Similarities in the HR decay pattern between our two groups indicate an appropriate paced HR recovery profile (Figure 5). The profile of HR decay has been shown to influence hemodynamics during exercise recovery [33] however, recovery algorithms are not regulated in the same manner as rate responsive algorithms. In the present study, following MCLR pacing and upon termination of movement, there was a progressive decay in pacing at a rate of 1 beat.min^-1^ every two cardiac cycles. This slope factor was non-physiologic in nature and independent of myocardial contractility. Lau et al [33] demonstrated that a compensatory SV response occurs during early recovery to ensure adequate Q during an abrupt HR decay (immediate decrease to 70 beats.min^-1^) versus a modulated decay (progressive decrease to 70 beats.min^-1^) following maximal exercise (at 130 beats.min^-1^). Thus, the similarities in Q and O_2_ Pulse responses at minute three of recovery for our study patients (see Table II) suggests an appropriate SV response that may in part have been mediated by the rate decay. The same study by Lau et al [33] also demonstrated that an abrupt HR rate decay significantly increased SBP and decreased DBP relative to a modulated HR decay. In the present study, similarities in arterial pressure responses during recovery (see Table II) would indicate that inappropriate baroreceptor stimulation did not occur, further implying that the initial HR rate decay appeared to be physiologically appropriate [33].

Dynamics of myocardial contractile force reflect control signals from the circulatory centres. Because the right ventricular impedance sensor driven pacemaker is theorized to use the intrinsic regulatory mechanism of the circulatory centres to control the rate response of the pacemaker, this method of rate response is considered to be closed loop. As a consequence of the internal feedback loop, the Inos^2+^ CLS system is expected to provide an appropriate rate response to exercise, as well as to account for each individual’s disease state and physical condition [34]. Even in those patients with dysfunctional sinus nodes or electrical pathways, neural control mechanisms attempt to control Q by varying inotropy. Earlier versions of this pacemaker showed the rate response to be highly correlated with control groups during both physical and mental stress challanges [34-36]. The results of the present study indicate that the Inos^2+^ CLS pacemaker was efficacious during incremental exercise and it would further appear that the sensor responded appropriately to metabolic demand. However, the pacemaker was not equipped to report measured cardiac contractility and as such, precludes specific comments on this system’s responsiveness to cardiac dynamics and thus, the degree to which this truly was a closed loop system.

## Limitations

The use of the pacemaker’s programmed settings for the MCLR in place of age-predicted maximum HR in the formulas may have affected the results. The accuracy of the use of age-predicted maximum HR in an older or paced population has been questioned by others [21-23] and may have been responsible for the lower than predicted MET levels. It is uncertain whether similar results would be have observed if the MCLR was set at each patient’s age-predicted maximum HR. Evaluating the rate-responsiveness of a pacemaker from rest to maximal exercise requires that the patient exercise to maximal capacity. Although our study patients were encouraged to exercise as long as they could, and all reached anaerobic threshold, we cannot be positive that all were motivated enough to achieve their maximal exercise workload.

The pacemakers were programmed to elicit 100% ventricular pacing throughout exercise, however, it was not possible to ensure 100% atrial pacing, as some patients were not completely dependent on the pacemaker. To address this limitation only paced HR values were included in the data analysis. The pacemaker programming of BR and MCLR was tailored to each patient. The purpose of this programming strategy was to closely mimic the clinical application. As a result the MCLR was typically set lower than 100% of maximum HR and the BR was set according to physician recommendations, in keeping with typical programming practices. The algorithm responds as a slope within these lower and upper values, and as such, the responses observed may have been slightly blunted compared to what may have been observed with MCLR programmed at 100% of maximum HR. However, more aggressive programming of upper rate limits is not the norm, thus, the response of the pacemaker was studied within practically applicable limits. Furthermore, the algorithm works such that it moderates the slope factor of the rate response according to the degree of responsiveness of the individual’s myocardial contractility change.

In summary, the results of the present study indicate the right ventricular impedance sensor driven pacemaker delivered pacing rates that resulted in overall cardiovascular responses comparable to that of healthy individuals throughout incremental exercise. Further, the pacemaker provided appropriate rate response up to the anaerobic threshold. Additionally, the rate decay algorithm appeared to result in physiologically appropriate hemodynamics during the initial minutes of recovery.

## Figures and Tables

**Table I:**
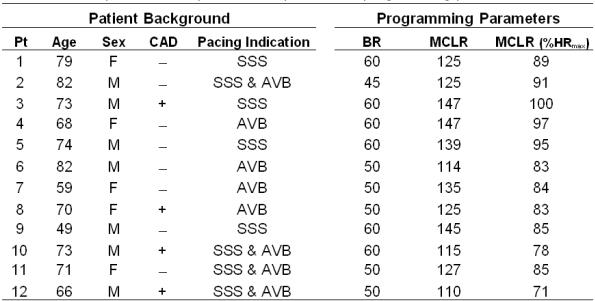
Descriptive data for patients and pacemaker programming parameters

Pt = patient number, age (years), F = female, M = male, CAD = coronary artery disease (+ = yes; - = no), SSS = sick sinus syndrome, AVB = atrioventricular block, BR = basic rate (programmed lowest pacing rate, beats.min^-1^), MCLR = maximum closed loop rate (programmed maximum upper pacing rate, beats.min^-1^), %HR_max_ = percentage of age predicted maximal heart rate

**Table II:**
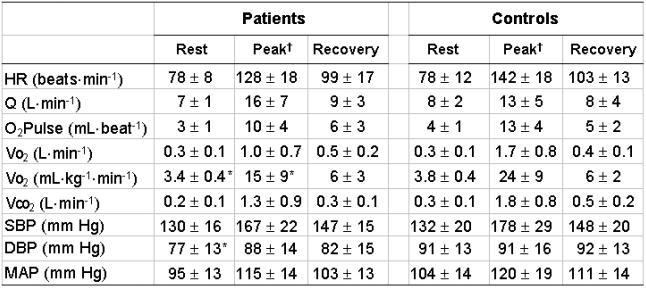
Physiologic measurements observed at rest, peak exercise and minute three of recovery for paced patients and healthy controls

Data are expressed as mean values ± standard deviation. HR = heart rate, Q= cardiac output, O_2_ Pulse = oxygen pulse, Vo_2_ = oxygen uptake, Vco_2_ = carbon dioxide production, SBP = systolic blood pressure, DBP = diastolic blood pressure and MAP = mean arterial pressure. †All variables decreased significantly (p < 0.05) from peak exercise to minute three of recovery except DBP. *Significantly different (p < 0.05) versus controls.
